# Brexpiprazole in India and the United States: A Comparative Review of Regulatory Status, Clinical Evidence, Market Access, and Evidence Gaps

**DOI:** 10.7759/cureus.117032

**Published:** 2026-09-27

**Authors:** Natasha Gill, Supreet K Sohi, Neena Bhatti, Girish Joseph, Pallavi Abhilasha

**Affiliations:** 1 Psychiatry, New York Medical College, Saint Clare’s Health, Denville, USA; 2 Psychiatry, New York Medical College, St. Mary’s General Hospital, Passaic, USA; 3 Internal Medicine, Government Medical College, Patiala, IND; 4 Pharmacology and Therapeutics, Christian Medical College & Hospital, Ludhiana, IND; 5 Psychiatry, Christian Medical College & Hospital, Ludhiana, IND

**Keywords:** atypical antipsychotics, brexpiprazole, india, major depressive disorder, market access, pharmacoeconomics, real-world evidence, regulatory approval, schizophrenia, united states

## Abstract

Brexpiprazole is a serotonin-dopamine activity modulator first cleared by the United States Food and Drug Administration (FDA) in 2015 for adults with schizophrenia and as an adjunct to antidepressants in major depressive disorder (MDD); later authorizations extended its use to adolescent schizophrenia and to agitation arising in dementia due to Alzheimer's disease. Over the past decade, randomized controlled trials, long-term open-label extensions, and analyses of real-world claims have converged on a consistent efficacy profile and a tolerability profile that has generally been favorable, although direct head-to-head comparisons with other second-generation agents remain limited. The evidence base and access environment differ markedly between the two countries. In the United States, brexpiprazole is an established product with an extensive clinical evidence base supplemented by real-world claims, utilization, safety, and economic analyses. In contrast, India has only recently received regulatory approval for brexpiprazole, and brexpiprazole-specific clinical, real-world utilization, pricing, reimbursement, and pharmacoeconomic data remain limited. This narrative review consolidates the pharmacology, clinical-trial findings, safety record, and health-economic data on brexpiprazole and contrasts the mature evidence environment of the United States with the emerging one in India. The principal Indian evidence gap is therefore no longer regulatory recognition but the generation of local data on availability, affordability, utilization, effectiveness, and pharmacoeconomic value. Framed as a collaboration between Christian Medical College and Hospital, Ludhiana, and New York Medical College at St Clare’s and St Mary’s, New Jersey, the review aims to support the equitable and evidence-based uptake of newer antipsychotics in emerging settings.

## Introduction and background

Schizophrenia is a chronic and frequently disabling psychotic disorder that affects approximately 27 million people worldwide, with a global prevalence of about 0.29% [1]. Antipsychotic medicines constitute the mainstay of pharmacological treatment for schizophrenia, with the choice of agent guided by efficacy, adverse-effect profile, previous treatment response, patient preferences, and clinical context [2]. Although effective, these agents differ in their tolerability profiles, with weight gain, metabolic abnormalities, sedation, akathisia, hyperprolactinemia, and QTc prolongation representing important treatment considerations [2,3]. Such adverse effects may influence adherence and persistence with treatment, while relapse contributes substantially to hospitalization and healthcare expenditure [4]. Consequently, selection of an antipsychotic has implications not only for symptom control and tolerability but also for healthcare utilization and treatment costs.

Brexpiprazole (OPC-34712) is a serotonin-dopamine activity modulator that received United States Food and Drug Administration (FDA) approval in July 2015 for the treatment of adults with schizophrenia and as adjunctive therapy to antidepressants for major depressive disorder (MDD) [3]. Subsequent regulatory expansion included schizophrenia in adolescents and agitation associated with dementia due to Alzheimer's disease [3]. Serotonin-dopamine activity modulation refers to the drug’s effects on dopamine and serotonin receptor signaling. As a partial agonist, brexpiprazole can activate a receptor but produces a less than maximal receptor response, with its overall effect depending on the receptor and surrounding signaling environment [4,5]. Its clinical development has generated evidence from randomized controlled trials, longer-term studies, systematic reviews, and real-world claims analyses, providing a substantial evidence base for evaluating its clinical efficacy, safety, utilization, and economic implications [3-6]. Detailed consideration of its receptor pharmacology and pharmacokinetic characteristics is presented separately in the Pharmacology and Mechanism of Action section.

The United States (US) and India were selected for comparison because they represent different stages of the evidence and access pathway for brexpiprazole. The US provides a mature comparator in which brexpiprazole has accumulated regulatory, clinical, utilization, safety, and economic evidence over more than a decade [4,5,7,8]. In contrast, India received regulatory approval for brexpiprazole in 2026, with subsequent approvals of finished formulations for domestic manufacturers [9]. However, brexpiprazole-specific Indian evidence regarding availability, affordability, utilization, safety, effectiveness, pricing, reimbursement, and pharmacoeconomic value remains limited. Thus, the comparison is not intended to imply equivalent clinical or real-world evidence from the two countries; rather, it examines how an established US evidence environment contrasts with the early post-approval Indian setting in terms of regulatory status, evidence maturity, market access, and health-system context. The US was chosen as the comparator because it provides the most extensive and mature brexpiprazole-specific evidence base, including regulatory, clinical, real-world, and economic data [4,5,7-9]. The comparison therefore focuses on differences in evidence maturity and healthcare context rather than on regulatory or generic-market similarity between the two countries.

This distinction is particularly relevant because regulatory approval does not necessarily establish widespread commercial availability, reimbursement, or routine utilization. In the US, brexpiprazole is an established marketed product for which claims-based studies have examined healthcare utilization and costs [4,7,9]. In India, the 2026 regulatory approval represents an important regulatory milestone, but the extent of commercial launch, pricing, distribution, reimbursement, prescribing, and locally generated outcomes remains to be characterized [10]. The Indian setting therefore provides an opportunity to identify the types of clinical, utilization, safety, and pharmacoeconomic evidence that will be needed as post-approval experience develops.

Against this background, this narrative review aimed to: (1) summarize the pharmacological properties and clinical efficacy of brexpiprazole across its major indications; (2) examine its safety, tolerability, and real-world utilization; (3) describe its regulatory status, market access, and health-economic evidence in the US and India; and (4) identify evidence gaps relevant to the emerging use of brexpiprazole in India. Particular emphasis was placed on distinguishing the established US evidence base from the currently limited Indian evidence and on identifying areas in which US experience may provide a framework for future Indian research.

## Review

Literature search strategy

A narrative literature search was conducted in PubMed/MEDLINE, Embase, Scopus, and Google Scholar from January 2014 to September 2026. The search strategy combined the terms “brexpiprazole,” “Rexulti,” and “OPC-34712” with terms related to the principal clinical indications and review domains, including “schizophrenia,” “major depressive disorder,” “Alzheimer disease agitation,” “adolescents,” “safety,” “tolerability,” “pharmacokinetics,” “health economics,” “cost,” “healthcare utilization,” “market access,” “regulatory approval,” “India,” and “United States.” Boolean operators AND and OR were used to combine concepts. Representative combinations included (brexpiprazole OR Rexulti OR OPC-34712) AND (schizophrenia OR “major depressive disorder” OR “Alzheimer disease agitation”) AND (safety OR efficacy OR pharmacokinetics OR cost OR “healthcare utilization” OR “market access”), with additional country-specific combinations using (India OR “United States”). The complete database-specific search strategies, including search terms, Boolean operators, filters, and search dates, are provided in the Appendices (Supplementary Table S1).

Regulatory information was searched separately in the official databases and publications of the US FDA, including prescribing information and the Orange Book, and the Central Drugs Standard Control Organisation (CDSCO) of India. Reference lists of relevant systematic reviews, meta-analyses, narrative reviews, pivotal clinical trials, and regulatory documents were also screened to identify additional relevant sources.

Sources were considered eligible when they provided information directly relevant to brexpiprazole's pharmacology, mechanism of action, clinical efficacy, safety, regulatory status, market access, healthcare utilization, pharmacoeconomics, or real-world use in the US or India. Randomized controlled trials, observational and claims-based studies, systematic reviews, meta-analyses, pharmacological studies, regulatory documents, and relevant epidemiological or health-system reports were considered. Publications without substantive relevance to brexpiprazole, duplicate reports, and sources that did not contribute to the objectives of the review were excluded. Sources were not excluded solely on the basis of publication date or publication type when they provided information relevant to the review objectives.

Three reviewers independently assessed the identified literature for relevance using a narrative screening approach. Potentially relevant titles and abstracts were screened, followed by full-text assessment where appropriate. Any disagreements regarding inclusion, relevance, or evidence interpretation were discussed among the three reviewers and resolved by consensus. Duplicate records were removed before final source selection. Source prioritization was based primarily on relevance to the review objectives, directness of evidence, methodological appropriateness, and contribution to the predefined review domains. Priority was given to primary clinical trials, official regulatory documents, systematic reviews and meta-analyses, and relevant observational or claims-based studies. When multiple publications addressed substantially overlapping populations or questions, the source providing the most directly relevant or comprehensive evidence was prioritized to minimize redundancy. Sources were excluded when they were duplicates, lacked substantive relevance to brexpiprazole or the India-United States comparison, provided redundant information without additional contribution, or did not meet the predefined eligibility criteria.

Across the bibliographic databases and additional regulatory and official sources, 722 records were identified. After removal of 185 duplicate records, 537 records were screened, and 123 full-text sources were assessed for eligibility. Ninety full-text sources were excluded because they did not meet the predefined eligibility criteria or did not provide substantive information relevant to the objectives of the review. A total of 36 sources were included in the final narrative synthesis. A quantitative summary of the literature identification and screening process is provided in the Appendices (Supplementary Table S2).

The 36 included sources comprised eight randomized clinical trials, four systematic reviews or meta-analyses, six observational or claims-based studies, four economic analyses, three regulatory documents, and other relevant sources, including pharmacological studies, narrative reviews, epidemiological studies, clinical guidelines, and health-system reports. Because individual sources could contribute to more than one evidence category, these categories were not mutually exclusive. A detailed composition of the evidence base is provided in the Appendices (Supplementary Table S3). Because this was a narrative rather than systematic review, no formal risk-of-bias assessment, Preferred Reporting Items for Systematic Reviews and Meta-analyses (PRISMA) flow diagram, or quantitative meta-analysis were undertaken. The evidence was synthesized qualitatively according to pharmacology, clinical efficacy and safety, regulatory status, access, health economics, and real-world evidence.

Pharmacology and mechanism of action

Brexpiprazole is a quinolinone whose scaffold closely resembles that of aripiprazole, the principal difference being an added thiophene-bearing side chain; this modification lowers intrinsic D2 activity while raising potency at 5-HT1A and strengthening antagonism at 5-HT2A [4,6]. In vitro, it binds several targets in the sub-nanomolar range and combines potent antagonism at the α1B and α2C adrenoceptors with only intermediate affinity for histamine H1 (Table 1) [4,11]. In functional terms, it behaves as a partial agonist at dopamine D2 and serotonin 5-HT1A receptors and as an antagonist at 5-HT2A [4]. Brexpiprazole’s partial agonism at D2 receptors and activity at 5-HT1A and 5-HT2A receptors are considered important components of its pharmacological profile. These receptor-level properties have been proposed as possible contributors to its clinical effects, including antipsychotic efficacy and aspects of tolerability; however, such receptor-level associations should be regarded as mechanistic hypotheses rather than established causal relationships [4]. These receptor-level mechanisms are hypotheses about how the drug may act and should not be interpreted as direct evidence of clinical superiority over other agents; the comparatively moderate H1 affinity has similarly been offered as a partial explanation for the drug’s tolerability profile rather than as an established determinant of outcome (Figure 1) [4,12].

**Table 1:** Receptor-binding profile and proposed functional activity of brexpiprazole 5-HT: 5-hydroxytryptamine; α1B: adrenergic receptor; α2C: adrenergic receptor The receptor affinity values presented in this table were derived from published studies using different experimental assay systems and conditions. Differences in receptor preparations, assay methodology, and experimental conditions may affect the reported Ki values; therefore, these values are provided for contextual comparison and should not be interpreted as directly comparable quantitative measurements across studies. Compiled from references [4,11]

Receptor target	Affinity, Ki (~nM)	Functional action	Proposed clinical implications (hypothesis)
Dopamine D2	0.3	Partial agonist	May contribute to antipsychotic efficacy with a potentially low extrapyramidal and prolactin liability
Serotonin 5-HT1A	0.12	Partial agonist	Proposed role in antidepressant, anxiolytic, and procognitive effects; potentially reduced akathisia
Serotonin 5-HT2A	0.47	Antagonist	Potentially associated with reduced extrapyramidal symptoms and antipsychotic augmentation
Adrenergic α1B/α2C	Potent	Antagonist	May underlie differentiation from aripiprazole; possible mood and cognitive effects
Histamine H1	Moderate	Antagonist	May contribute to modest weight-gain and sedation liability

**Figure 1:**
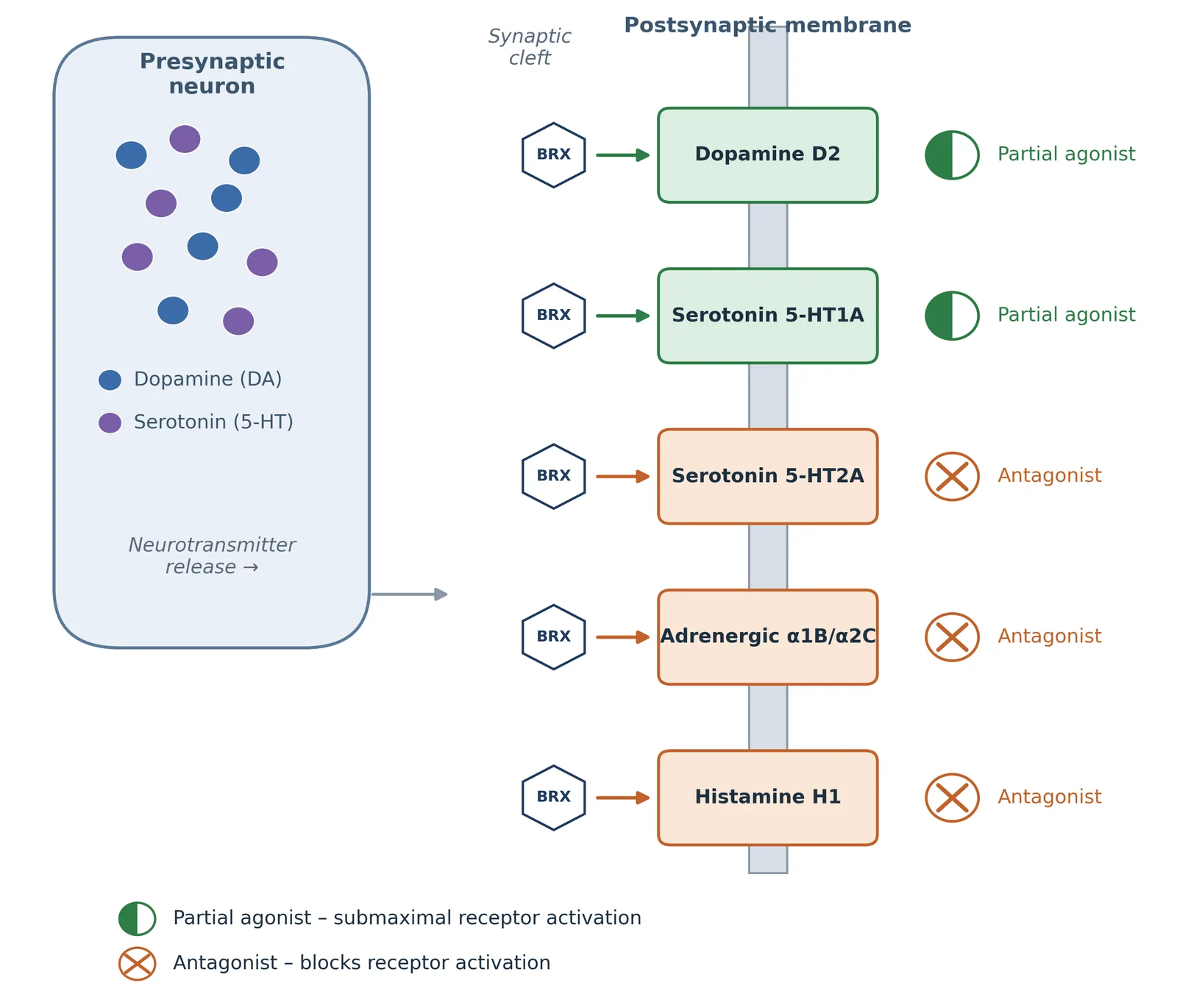
Schematic mechanism of action of brexpiprazole at the synapse BRZ: brexpiprazole; 5-HT: 5-hydroxytryptamine; α1B: adrenergic receptor; α2C: adrenergic receptor BRX engages five key receptors on the postsynaptic membrane, acting as a partial agonist (submaximal activation) at dopamine D2 and serotonin 5-HT1A receptors and as an antagonist (blocking activation) at serotonin 5-HT2A, adrenergic α1B/α2C, and histamine H1 receptors. Original artwork created by the authors using Python (Matplotlib), conceptually informed by the receptor-binding characterization of Maeda et al. and the icon-based schematic approach of Stahl; the figure depicts receptor-level pharmacology only and should not be read as evidence of a specific clinical effect

The receptor-level properties summarized in Table 1 describe pharmacological mechanisms and preclinical findings. Any proposed links between these receptor actions and clinical effects represent mechanistic hypotheses and should not be interpreted as clinically demonstrated benefits. The underlying receptor-binding characterization was first reported by Maeda et al. in preclinical in vitro and in vivo studies [12].

Brexpiprazole belongs to a lineage of dopamine partial agonists whose prototype, aripiprazole, introduced the serotonin-dopamine activity-modulator concept ahead of both brexpiprazole and cariprazine [6]. Relative to that predecessor, brexpiprazole was designed with reduced intrinsic D2 activity-positioned nearer the antagonist end of the partial-agonist spectrum while binding more tightly at 5-HT2A, 5-HT1A, and α1B, a configuration hypothesized to contribute to its pharmacological profile and potentially influence activation-related effects such as agitation and akathisia [6]. Because no head-to-head trials compare the two agents directly, this distinction rests on receptor-level data and indirect comparisons. A network meta-analysis of randomized trials found that short-term efficacy and tolerability in acute schizophrenia did not differ appreciably between aripiprazole and brexpiprazole, so any advantage in activation-related adverse events remains a plausible pharmacological hypothesis rather than an established clinical finding [6]. Frankel and Schwartz likewise frame these three dopamine partial agonists as differing chiefly in their intrinsic activity at D2 and their relative serotonergic and adrenergic affinities rather than in their core mechanism, a distinction Stahl originally summarized using qualitative receptor-binding icons and which is compared across the class in Figure 2 [13,14].

**Figure 2:**
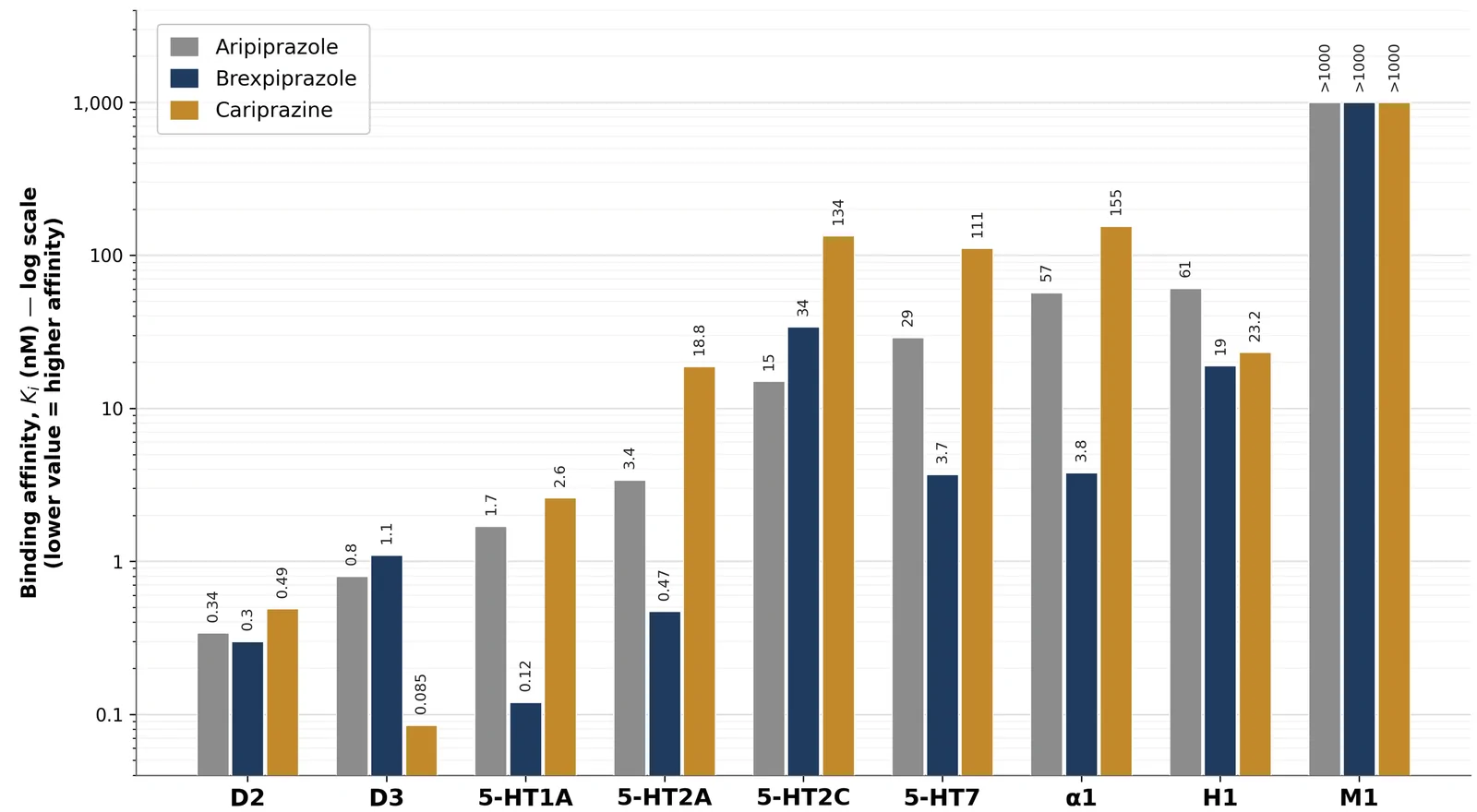
Comparative receptor-affinity profile of the three dopamine partial agonists (aripiprazole, brexpiprazole, and cariprazine) Comparative receptor-binding affinity expressed as Ki (nM) on a logarithmic scale, with lower values indicating greater binding affinity. Values are based on published receptor-binding data summarized by Frankel and Schwartz from US FDA prescribing information. Because Ki values may be derived from different experimental assay systems and conditions, they should be interpreted as contextual rather than directly equivalent quantitative comparisons. Receptor-binding affinity describes pharmacological properties and does not by itself establish clinical efficacy, safety, or clinical superiority. Figure created by the authors using Python (Matplotlib). Compiled from references [4,6,13,14]

Beyond the conventional monoamine-receptor framework, mechanistic work has begun to examine how brexpiprazole influences glial signaling. In cultured astrocytes, subchronic exposure at therapeutically relevant concentrations reduces expression of 5-HT1A and 5-HT7 receptors and attenuates downstream adenosine monophosphate (AMP)-activated protein kinase and extracellular signal-regulated kinase activity-a pattern consistent with a 5-HT7 inverse-agonist-like effect reported for this drug class [11]. These findings provide a potential biological rationale for antidepressant and metabolic effects, but these proposed mechanisms remain hypothetical and require further translational and clinical validation [11].

According to the FDA prescribing information, brexpiprazole is well-absorbed after oral administration and reaches steady state within approximately 10-12 days. Its prolonged plasma half-life results in relatively slow elimination, which may provide some pharmacokinetic buffering against fluctuations in plasma concentrations following an occasional missed dose; however, this is a pharmacokinetic inference and has not been established as a clinical adherence benefit (Table 2) [3]. Metabolism proceeds chiefly through the cytochrome P450 enzymes CYP2D6 and CYP3A4, yielding the active but less potent metabolite DM-3411, so dose reduction is advised in poor CYP2D6 metabolizers and when strong CYP2D6 or CYP3A4 inhibitors are co-administered [3]. Absorption is essentially unaffected by food, a practical advantage over agents such as lurasidone and ziprasidone, which require a calorie-specified meal, and the long half-life cushions the pharmacological impact of an occasional missed dose [3,4].

**Table 2:** Key pharmacokinetic parameters of brexpiprazole DM-3411: brexpiprazole S-oxide; CYP: cytochrome; FDA: Food and Drug Administration Values are taken from the FDA prescribing information and a supporting pharmacology review. Compiled from references [3,4]

Parameter	Value/characteristic
Oral bioavailability	~95%
Time to peak concentration (Tmax)	~4 hours
Plasma half-life	~91 hours
Time to steady state	10-12 days
Principal metabolism	CYP2D6 and CYP3A4 → DM-3411 (active, less potent)
Effect of food	No clinically meaningful effect on absorption
Dose adjustment advised	Poor CYP2D6 metabolizers; strong CYP2D6 or CYP3A4 inhibitors

Clinical efficacy in schizophrenia

The short-term efficacy of brexpiprazole in acute schizophrenia was established in fixed-dose, randomized, double-blind, placebo-controlled phase 3 studies. In the VECTOR trial, brexpiprazole 2 mg and 4 mg once daily produced statistically significant improvements from baseline in Positive and Negative Syndrome Scale (PANSS) total score relative to placebo [15]. In the BEACON trial, which tested 1, 2, and 4 mg daily, separation from placebo on the PANSS was demonstrated for the 4 mg arm [16]. Pooled meta-analytic data showed significant improvements in PANSS total scores with both brexpiprazole 2 mg/day (SMD: -0.30; 95% CI: -0.46 to -0.13) and 4 mg/day (SMD: -0.30; 95% CI: -0.43 to -0.17) versus placebo. For PANSS negative symptoms, the corresponding SMDs were -0.32 (95% CI: -0.51 to -0.13) for 2 mg/day and -0.30 (95% CI: -0.43 to -0.17) for 4 mg/day. For positive symptoms, the SMD was -0.17 (95% CI: −0.32 to -0.02) with 2 mg/day, whereas the 4 mg estimate was -0.18 (95% CI: −0.42 to 0.07) and was not statistically significant in the primary analysis [4,6]. In long-term open-label extensions, gains achieved during acute treatment were sustained or extended over 52 weeks, and a randomized-withdrawal maintenance study showed a lower relapse risk and a longer time to discontinuation than placebo [4].

Adolescent Schizophrenia and the Extrapolation Approval

Brexpiprazole was first approved by the FDA in 2015 for adults with schizophrenia and subsequently received an adolescent schizophrenia indication in 2021. The adolescent approval was based on extrapolation of adult efficacy data, supported by population pharmacokinetic modeling and pediatric safety data, rather than on a completed placebo-controlled efficacy trial in adolescents at the time of the regulatory decision [3,17]. This approach was intended to reduce placebo exposure in a vulnerable population and shorten the interval between adult and pediatric development. However, concerns were raised regarding the extent to which adult efficacy could be extrapolated to adolescents, particularly because some antipsychotics with established adult efficacy, including ziprasidone and asenapine, had previously failed to demonstrate separation from placebo in adolescent schizophrenia trials [17].

The regulatory sequence was subsequently followed by direct clinical evaluation in adolescents. A multicountry, randomized, double-blind, placebo-controlled phase 3 trial with an aripiprazole active reference enrolled 316 adolescents aged 13-17 years with schizophrenia across 10 countries [18]. At week six, brexpiprazole 2-4 mg/day produced a least-squares mean change in PANSS total score of -22.8 compared with -17.4 with placebo, corresponding to a treatment difference of -5.33 (95% CI: -9.55 to -1.10; p = 0.014) [18]. The aripiprazole reference provided evidence of assay sensitivity [18]. Safety findings were broadly consistent with the established profile, although the high placebo response and exclusion of some comorbid presentations should be considered when interpreting applicability to routine practice [18].

Adjunctive Use in MDD

A substantial proportion of patients with MDD do not achieve remission following an initial adequate antidepressant trial; in the Sequenced Treatment Alternatives to Relieve Depression (STAR*D) study, remission occurred in 36.8% of patients after the first treatment step [19]. In this context, inadequate benefit refers to failure to achieve remission despite an adequately dosed and sufficiently long treatment trial [19]. The short-term efficacy of adjunctive brexpiprazole was established in two pivotal six-week phase 3 randomized controlled trials in patients with an inadequate antidepressant response. In the PYXIS trial, adjunctive brexpiprazole 2 mg was separated from adjunctive placebo on the Montgomery-Åsberg Depression Rating Scale (MADRS) [20]. In the POLARIS trial, which evaluated 1 mg and 3 mg, the 3 mg dose showed significant benefit on the MADRS (Table 3) [21]. A systematic review of 11 randomized controlled trials enrolling about 5,300 participants, which weighed second-generation antipsychotic augmentation strategies in treatment-resistant depression, identified brexpiprazole and the aripiprazole-sertraline pairing as offering among the most consistent benefit, with brexpiprazole augmentation producing remission rates of roughly 22% to 31% and class-wide discontinuation for adverse events remaining low [22]. A more recent network meta-analysis of adjunctive antipsychotics in MDD, pooling 22 short-term studies in nearly 11,000 participants, reaffirmed the efficacy of brexpiprazole alongside aripiprazole, cariprazine, quetiapine extended release, and lumateperone [23].

**Table 3:** Summary of pivotal phase 3 trials of brexpiprazole PANSS: Positive and Negative Syndrome Scale; LS: least-squares; CI: confidence interval; MDD: major depressive disorder; MADRS: Montgomery-Åsberg Depression Rating Scale. Statistical significance refers to comparisons with placebo or adjunctive placebo as specified in the individual trials

Indication (trial)	Population	Dose	Key efficacy outcome
Adult schizophrenia (VECTOR) [16]	Adults, acute episode	2-4 mg/day	Significant PANSS total reduction vs placebo (2 and 4 mg)
Adult schizophrenia (BEACON) [17]	Adults, acute episode	1-4 mg/day	Significant PANSS reduction for 4 mg vs placebo
Adolescent schizophrenia (multicountry phase 3) [7]	316 adolescents, 13-17 yr	2-4 mg/day	LS mean PANSS change -22.8 vs -17.4; difference -5.33 (95% CI: -9.55 to -1.10; p = 0.014)
Adjunctive MDD (PYXIS) [20]	Adults, inadequate antidepressant response	2 mg/day	Significant MADRS improvement vs adjunctive placebo
Adjunctive MDD (POLARIS) [21]	Adults, inadequate antidepressant response	1-3 mg/day	Significant MADRS improvement for 3 mg vs adjunctive placebo
Adjunctive MDD maintenance (randomized withdrawal) [22]	Adults stabilized on antidepressant + brexpiprazole	Continue vs placebo	No significant difference in time to relapse; interpretation limited by design

A subsequent phase 3, double-blind, placebo-controlled, randomized-withdrawal study addressed maintenance. Patients stabilized on an antidepressant plus brexpiprazole were re-randomized either to continue the combination or to switch to placebo; time to relapse did not differ significantly between the arms, and a high proportion of stabilized patients in both groups remained relapse-free through the double-blind phase [24]. This result should be interpreted with the study design in mind: in randomized-withdrawal trials, low observed relapse rates and the characteristics of the stabilized population can limit the ability to detect a maintenance effect, so the finding does not by itself establish an absence of maintenance benefit [24]. The authors additionally noted that discontinuation of adjunctive brexpiprazole produced only minimal withdrawal effects, a feature attributed partly to the molecule’s long half-life [24]. The pivotal trials demonstrated statistically significant improvements in schizophrenia and MDD symptom measures compared with placebo, although the magnitude of benefit was generally modest [15,16,20,21]. In a network meta-analysis of antipsychotics for acute schizophrenia, brexpiprazole showed a standardized mean difference of -0.26 (95% CI: -0.39 to -0.12) versus placebo for overall symptom reduction. Differences between individual antipsychotics were generally small or uncertain, although some agents demonstrated greater efficacy in specific comparisons [25].

Safety and Tolerability

An integrated analysis that pooled short-term controlled studies with two 52-week open-label studies delineated the safety profile of brexpiprazole in schizophrenia [4]. In the short-term dataset, no treatment-emergent adverse event occurred at a frequency of at least 5% and double that of placebo among patients on 2 mg to 4 mg [4]. Akathisia was relatively infrequent in both the short-term (about 6%) and long-term (about 5%) settings, as was sedation, and the average weight increase was roughly one kilogram in each; shifts in lipids, glucose, other metabolic indices, electrocardiographic measures, and prolactin were generally minor across the studies [4]. Estimated numbers needed to harm for akathisia, sedation, and clinically relevant weight gain have been reported as comparing favorably with published values for aripiprazole, risperidone, olanzapine, and quetiapine, although these are indirect comparisons rather than head-to-head data (Table 4) [4,6].

**Table 4:** Selected tolerability findings reported for brexpiprazole and aripiprazole Adverse-event frequencies are reported where comparable quantitative data were available. Direct numerical comparisons between brexpiprazole and aripiprazole should be interpreted cautiously because available evidence is derived predominantly from separate trials and indirect/network comparisons with differences in populations, study duration, dosing, and outcome definitions. Compiled from references [4,6]

Adverse effect	Brexpiprazole	Aripiprazole	Interpretation/evidence limitation
Akathisia	~6% in short-term schizophrenia studies; ~5% in longer-term studies	Reported with aripiprazole; direct comparison varies by study	No significant short-term difference demonstrated in available network evidence; indirect comparison
Somnolence/sedation	Reported; frequency varies by indication and study	Reported; frequency varies by indication and study	Numerical cross-drug comparison is limited by differences in study populations and designs
Weight gain	Mean increase approximately 1 kg in short-term studies	Generally small mean weight change in short-term studies	Magnitudes vary across trials; indirect comparisons should be interpreted cautiously
Prolactin elevation	Generally small changes in clinical trials	Generally small changes in clinical trials	No direct head-to-head quantitative comparison established
QTc prolongation	No clinically meaningful QTc effect identified in clinical development	No clinically meaningful QTc effect identified in clinical development	Cross-study numerical comparison is not appropriate because ECG methods and populations differ

The tolerability profile is best described as generally favorable rather than uniformly benign. A broad review spanning schizophrenia and mood disorders reported that most adverse events are mild to moderate and that the risk of metabolic syndrome is low, while still noting a measurable, histamine-mediated tendency toward modest weight gain of about one kilogram at therapeutic doses [4]. Weight gain, somnolence, and akathisia are recognized class-related effects that warrant routine metabolic and clinical monitoring, and the compound carries the antipsychotic class boxed warning for increased mortality in elderly patients with dementia-related psychosis [3,26]. In adolescents, a long-term open-label study reported a safety profile broadly matching that of adults, with somnolence, headache, and weight increase the most common treatment-emergent events; once expected growth was accounted for, clinically meaningful weight gain affected about one in five adolescents, close to the adult proportion, and these pediatric findings informed the extrapolation-based approval [17,27].

In 2023, brexpiprazole became the first agent approved specifically for agitation associated with Alzheimer's disease. Real-world observational data have reported an association between brexpiprazole use and mortality outcomes; however, these findings do not establish a causal mortality benefit because of potential residual confounding and differences between observational and instrumental-variable analyses. The FDA boxed warning regarding increased mortality in elderly patients with dementia-related psychosis should also be considered when interpreting these findings [10,28]. Real-world evidence has begun to place that warning in context. A retrospective study using a complete sample of United States Medicare claims from 2014 to 2023 compared new users of brexpiprazole with matched new users of aripiprazole among people living with dementia [10]. Six-month mortality was not elevated with brexpiprazole; in matched logistic regression it was in fact lower (odds ratio: 0.49; 95% CI: 0.37-0.65), while emergency-department visits and hospitalizations did not differ significantly between the drugs [10]. When possible unobserved confounding was addressed through an instrumental-variable, two-stage least-squares analysis, the mortality difference between the agents was no longer statistically significant, indicating no excess risk for brexpiprazole relative to aripiprazole rather than a protective effect [10]. Because observational mortality analyses cannot rule out residual or unmeasured confounding, the investigators also used an instrumental-variable, two-stage least-squares analysis as a sensitivity analysis. This approach was used to assess whether the observed mortality association was robust to potential unmeasured confounding. The instrumental-variable estimate was interpreted alongside the primary observational analysis rather than as definitive evidence of a causal effect [10].

Real-World Effectiveness and Complex Populations

Beyond the registration trials, a growing body of observational and real-world data has examined brexpiprazole in the mixed populations encountered in routine care, where adherence and tolerability often shape long-term outcome [29]. A narrative synthesis of work published between 2020 and 2026 reported that available observational and real-world studies generally describe improvements in positive and negative symptoms together with a favorable tolerability profile-little activation, low sedation, reduced extrapyramidal risk, and modest cardiometabolic burden-features that have led authors to consider it for patients requiring long-term treatment and polypharmacological management, including those with prominent negative symptoms or with comorbidities that complicate adherence [29]. Because these reports are largely uncontrolled, they describe associations rather than establish efficacy in the sense provided by randomized trials. Nonetheless, real-world tolerability is relevant to the health-economic and access issues that follow, since durable adherence is an important determinant of relapse risk and, in turn, of the hospitalization costs that dominate the economics of schizophrenia [5,29].

Health-Economic Evidence and Real-World Utilization

For this review, economic evidence was considered in two categories: (1) healthcare-resource utilization and cost studies, which assess observed healthcare use and expenditures in real-world populations, and (2) formal pharmacoeconomic evaluations, including cost-effectiveness, cost-utility, and budget-impact analyses. The brexpiprazole evidence identified in this review predominantly comprises retrospective claims-based utilization and cost studies rather than formal cost-effectiveness, cost-utility, or budget-impact analyses [5,8,30,31]. Accordingly, the findings below are interpreted as evidence of healthcare utilization and expenditure rather than as evidence of economic value or cost-effectiveness.

Because hospitalization contributes substantially to the cost of schizophrenia, real-world utilization data provide relevant economic information. A retrospective cohort study using two large United States claims databases compared brexpiprazole with seven other oral atypical antipsychotics in adults with schizophrenia [5]. After adjustment for baseline characteristics and medication adherence, all-cause hospitalizations and total and medical costs did not differ significantly among the treatment groups; however, brexpiprazole-treated patients had the lowest mean psychiatric costs, while paliperidone- and quetiapine-treated patients had significantly more psychiatric hospitalizations per year than brexpiprazole-treated patients [5]. These findings represent observed associations in claims data and should not be interpreted as demonstrating that brexpiprazole causes lower healthcare costs.

In MDD, retrospective commercial claims analyses examined both comparative utilization and the timing of adjunctive brexpiprazole initiation [8,30,31]. One real-world comparison of adjunctive brexpiprazole with extended-release quetiapine reported lower all-cause hospitalization (6.6% vs. 12.5%) and emergency-department visits (17.0% vs. 27.5%), together with lower mean medical costs (US$6,421 vs. US$8,545); higher pharmacy costs offset these differences, resulting in no significant difference in total healthcare costs [8]. In another analysis, patients who initiated brexpiprazole more than 12 months after starting an antidepressant had higher MDD-specific outpatient and total costs and more outpatient visits than those initiating within the first two months [30]. These findings suggest an association between treatment timing and healthcare-resource use, although residual confounding and treatment-selection effects cannot be excluded [30]. Additional claims-based evidence reported differences in psychiatric costs between adjunctive brexpiprazole and other antipsychotic treatment groups [31].

Comparative Access: The United States and India

In the United States, brexpiprazole is FDA-approved, marketed as Rexulti, and available within commercial, Medicare, and Medicaid payer systems, with coverage varying conditions that have made possible the claims-based utilization studies described above [3,5,8,30]. The FDA approved a generic brexpiprazole (first abbreviated new drug application approval in January 2025), but patent protections have delayed generic market entry, so branded product availability continues to shape current United States acquisition costs; regulatory approval of a generic and its commercial launch are distinct milestones (Figure 3) [32]. Within this mature market, the clinical questions center on comparative effectiveness, tolerability, and timing of initiation rather than on availability as such.

**Figure 3:**
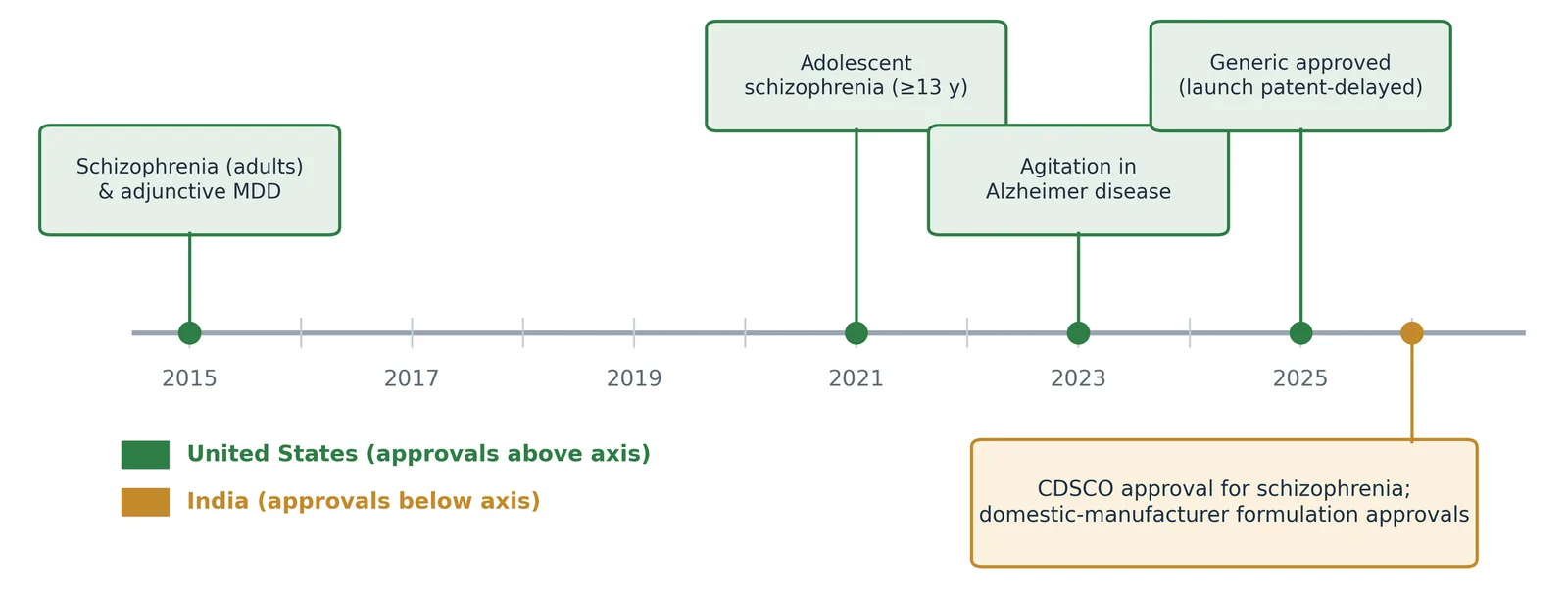
Regulatory-approval timeline for brexpiprazole in the United States (approvals shown above the axis) and India (below) MDD: major depressive disorder The United States milestones span schizophrenia and adjunctive major depressive disorder (2015), adolescent schizophrenia (2021), agitation in Alzheimer's disease (2023), and generic approval (2025); the Indian regulator approved the drug for schizophrenia in 2026, with domestic-manufacturer formulation approvals the same year. Regulatory approval denotes market entry rather than confirmed commercial launch. Compiled from references [5,11,30] Image credit: Created by Sohi, Joseph, and Bhatti using Canva (Canva Inc., Perth, Australia)

India has recently entered a new phase of brexpiprazole access. CDSCO approved brexpiprazole tablets in strengths of 0.25, 0.5, 1, 2, 3, and 4 mg for the treatment of schizophrenia in February 2026, with subsequent records documenting finished-formulation approvals during 2026 [9]. However, unlike the United States, India currently has limited brexpiprazole-specific published evidence on clinical use, safety, effectiveness, prescribing patterns, utilization, pricing, reimbursement, and pharmacoeconomic outcomes. Accordingly, the Indian component of this review is primarily regulatory and contextual rather than an assessment of established commercial access or real-world utilization [9]. Regulatory approval should not be interpreted as evidence of established market access. In the absence of reliable data on current retail launch, pricing, distribution, reimbursement, and prescribing patterns in India, this review assesses market access primarily from the perspective of regulatory status and the availability of evidence relevant to future access, rather than making claims about actual commercial availability or utilization. Before this approval, individual access depended on importation mechanisms, but that historical pathway should be distinguished from the current post-approval environment [9]. The clinical context in which this access is unfolding is demanding. Nationally representative estimates place the lifetime prevalence of schizophrenia spectrum disorders at about 1.4% and the current prevalence at 0.4%, alongside a treatment gap near 72% for current cases-meaning that most affected individuals receive no formal care [33]. India had an estimated 0.75 psychiatrists per 100,000 population in 2021, according to the Government of India Economic Survey 2023-24, which identified this as substantially below the WHO-referenced level of 3 psychiatrists per 100,000 population [34], and India continues to have substantial out-of-pocket health expenditure, which may pose an important barrier to access to newer medicines (Figure 4, Table 5) [35]. 

**Figure 4:**
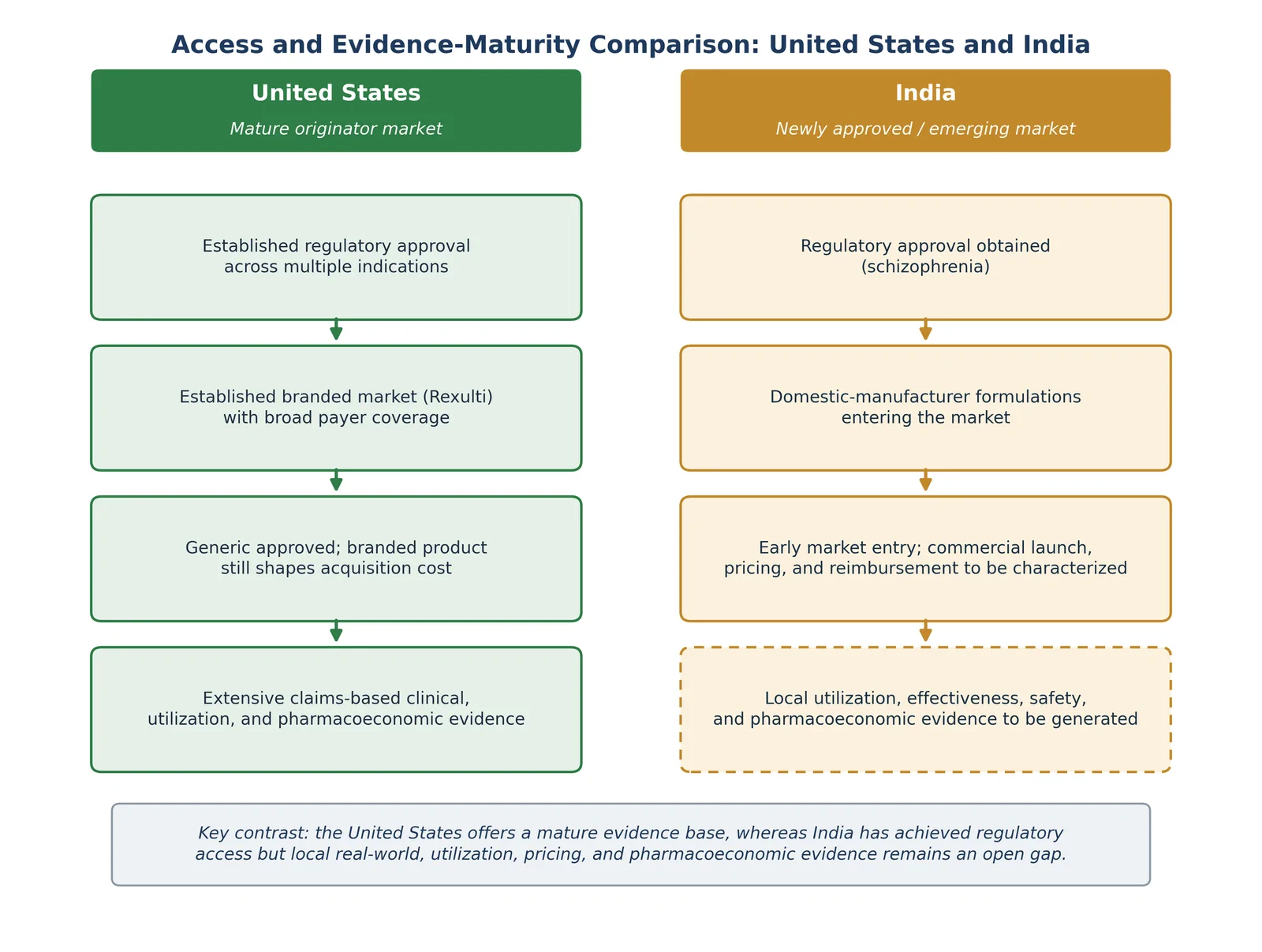
Access and evidence-maturity comparison for brexpiprazole in the United States and India The figure contrasts the stage of market and evidence development rather than chronology Image credit: Created by Abhilasha and Sohi using Canva (Canva Inc., Perth, Australia)

**Table 5:** Comparative regulatory, access, and evidence environment for brexpiprazole in the United States and India FDA: United States Food and Drug Administration; CDSCO: Central Drugs Standard Control Organization; MDD: major depressive disorder United States entries are drawn from FDA sources [3,32], and United States claims studies [5,8,30]; Indian entries are drawn from CDSCO regulatory records [9] and Indian mental-health epidemiology, workforce, and health-financing sources [33,35,36]

Dimension	United States	India
Regulatory status	FDA-approved	CDSCO-approved (2026)
Approved indication(s) relevant to this comparison	Schizophrenia, MDD augmentation, Alzheimer disease agitation	Schizophrenia
Market status	Established branded market; generic approved	Newly approved/emerging market
Commercial availability	Established	Requires verification of current commercial launch
Access	Prescription and pharmacy dispensing with payer variation	Prescription-based access; pricing and distribution evolving
Reimbursement	Commercial and public payer systems	Local reimbursement and coverage data limited
Real-world evidence	Extensive claims-based evidence	Limited/no substantial Indian utilization evidence identified
Key evidence gap	Comparative effectiveness and cost optimization	Local effectiveness, safety, affordability, utilization, and pharmacoeconomic evidence

Read together, these two settings illustrate a transition rather than a simple presence-versus-absence contrast. The American clinician chooses from an extensive menu of second-generation drugs partly on the basis of tolerability nuances and cost-timing considerations, whereas the Indian clinician has, until very recently, worked within a more limited range of registered antipsychotic treatment options compared with the broader range of agents and indications available in the United States. Comparative real-world evidence generated in the United States, such as the observation that antipsychotic treatment groups differ in psychiatric hospitalization and cost, may have limited immediate transferability to India, yet it provides a template for the kind of utilization and pharmacoeconomic evaluation that could, in principle, inform Indian formulary, pricing, and prescribing decisions now that the drug is approved [5,30]. A collaborative, cross-continental research agenda of the kind embodied in the partnership between Christian Medical College and Hospital, Ludhiana, and New York Medical College at St. Clare’s and St. Mary’s, New Jersey, is well-placed to help generate that locally relevant evidence.

Comparative Clinical and Health-System Implications

The contrast between the United States and India extends beyond regulatory timing to the maturity of the evidence and healthcare infrastructure supporting treatment decisions. In the United States, brexpiprazole has accumulated evidence across randomized clinical trials, long-term studies, claims-based analyses, and healthcare-resource utilization studies. Regulatory expansion has also resulted in indications spanning adult and adolescent schizophrenia, adjunctive treatment of MDD, and agitation associated with Alzheimer's disease [3,7,18,20,21,28]. In India, the regulatory pathway is substantially more recent. CDSCO approved brexpiprazole tablets for schizophrenia in 2026, followed by finished-formulation approvals for domestic manufacturers [9]. Thus, the immediate Indian evidence priority is not replication of the entire United States evidence base but generation of locally relevant evidence addressing effectiveness, safety, affordability, prescribing patterns, adherence, and healthcare utilization.

The economic implications are similarly context-dependent. United States claims studies suggest that brexpiprazole may be associated with differences in psychiatric healthcare utilization and medical costs compared with selected atypical antipsychotics, although these findings are observational and cannot establish causality [5,8,30,31]. In India, the interpretation of such evidence requires caution because medicine acquisition, insurance coverage, reimbursement mechanisms, healthcare-seeking behavior, and out-of-pocket expenditure differ substantially from the United States. Consequently, United States pharmacoeconomic estimates should be regarded as informative benchmarks rather than directly transferable cost-effectiveness estimates for Indian practice.

From a clinical perspective, brexpiprazole should be considered as one option within the broader antipsychotic treatment pathway rather than as a universally preferred agent. Clinicians should individualize treatment according to indication, previous treatment response, adverse-effect susceptibility, comorbidities, drug interactions, adherence considerations, and affordability. The long elimination half-life and relatively favorable tolerability profile may be useful considerations in selected patients, but these potential advantages should be balanced against acquisition cost and the limited availability of India-specific comparative effectiveness data [3,4,6].

For health systems, the United States experience demonstrates the value of linking regulatory approval with post-marketing surveillance, claims-based utilization research, and pharmacoeconomic evaluation. In India, the recent regulatory approval provides an opportunity to establish prospective and real-world evidence from the outset. Priority areas include comparative effectiveness against commonly used antipsychotics, real-world safety monitoring, adherence and persistence, patient out-of-pocket expenditure, availability across public and private sectors, and cost-effectiveness analyses using Indian healthcare costs and treatment pathways. Such evidence could assist clinicians, institutional formularies, payers, and policymakers in determining where brexpiprazole provides meaningful clinical or health-system value.

Limitations

Several caveats qualify how the literature reviewed here should be read. Much of the efficacy evidence comes from industry-sponsored trials whose restrictive entry criteria limit generalizability to real-world, comorbid populations. Industry sponsorship may also introduce potential publication and sponsor bias, including selective publication or reporting of favorable findings, influence on study design or comparator selection, and emphasis on selected efficacy or safety endpoints. Although the present review did not formally assess these biases, they should be considered when interpreting the available evidence. Comparisons between brexpiprazole and aripiprazole rest largely on receptor-binding data and indirect, cross-study contrasts rather than head-to-head trials; where such comparisons have been synthesized quantitatively, no significant short-term differences emerged, so claims of relative tolerability advantage remain provisional [6]. The real-world utilization and safety studies, valuable as they are, draw on administrative claims that are prone to coding error and residual confounding and that cannot capture illness severity or the clinical reasoning behind treatment choice [5,8,10,30]. The adolescent evidence, though now anchored by a placebo-controlled trial, was marked by a high placebo response and, for long-term safety, depends on an open-label study lacking an active comparator [18,27]. The observational and narrative real-world literature is heterogeneous and largely uncontrolled, and the treatment-resistant depression synthesis combined trials of differing designs and definitions [22,29]. As this was a narrative review rather than a systematic review, formal assessment of publication bias, selective outcome reporting, and sponsor-related bias was not undertaken, and these sources of bias cannot be excluded. The literature was identified and prioritized according to relevance to the review objectives, and therefore some potentially relevant studies may not have been included. Finally, and most pertinent to this review’s comparative aim, Indian real-world, utilization, pricing, and pharmacoeconomic data on brexpiprazole are almost entirely lacking, and the Indian regulatory and commercial landscape is evolving rapidly following the 2026 approval; information on commercial launch, pricing, reimbursement, and utilization may therefore change over time, and the comparisons drawn here are necessarily pitched at the level of health-system access and evidence maturity rather than head-to-head local outcomes [9].

Future Directions

Future research should focus on generating India-specific evidence as brexpiprazole transitions from regulatory approval to routine clinical use. Prospective observational studies and pragmatic comparative-effectiveness studies should evaluate prescribing patterns, treatment persistence, adherence, adverse effects, and patient-reported outcomes in routine psychiatric practice. Health-economic studies should incorporate Indian drug prices, healthcare utilization, hospitalization costs, out-of-pocket expenditure, and differences between public and private healthcare delivery. Post-marketing pharmacovigilance should also characterize the safety profile in Indian populations, including patients with multimorbidity and concomitant medications who may be underrepresented in registration trials. Finally, comparative research across India and the US could help determine which findings are transferable across health systems and which require locally adapted treatment and economic models.

## Conclusions

Brexpiprazole has accumulated a substantial evidence base for schizophrenia and for the adjunctive treatment of major depressive disorder, with additional evidence supporting its use in adolescent schizophrenia and in agitation associated with Alzheimer's disease. The comparison between the United States and India illustrates an important transition in access rather than a simple contrast between availability and absence: the United States represents a mature clinical and real-world evidence environment, whereas India is entering an emerging phase following recent regulatory approval. The principal evidence gap in India is, therefore, no longer regulatory recognition alone, but the generation of local data on availability, affordability, utilization, safety, effectiveness, and pharmacoeconomic value.

## Appendices

Supplementary Table 1

Methodological Documentation of Literature Search, Evidence Selection, and Evidence-Base Composition

**Table 6:** Detailed literature and regulatory search strategies

Database/source	Exact search date	Search string (keywords and Boolean operators)	Applied limits/filters
PubMed/MEDLINE	August 26, 2026	("brexpiprazole"[Title/Abstract] OR "Rexulti"[Title/Abstract] OR "OPC-34712"[Title/Abstract]) AND ("schizophrenia"[MeSH Terms] OR "schizophrenia"[Title/Abstract] OR "depressive disorder, major"[MeSH Terms] OR "major depressive disorder"[Title/Abstract] OR "Alzheimer Disease"[MeSH Terms] OR "Alzheimer disease agitation"[Title/Abstract] OR "adolescent"[MeSH Terms]) AND ("safety"[Title/Abstract] OR "tolerability"[Title/Abstract] OR "efficacy"[Title/Abstract] OR "pharmacokinetics"[MeSH Terms] OR "economics"[MeSH Terms] OR "cost"[Title/Abstract] OR "healthcare utilization"[Title/Abstract] OR "market access"[Title/Abstract] OR "drug approval"[MeSH Terms]) AND ("India"[MeSH Terms] OR "India"[Title/Abstract] OR "United States"[MeSH Terms] OR "United States"[Title/Abstract])	English language; Inception to August 2026
Embase	August 26, 2026	('brexpiprazole'/exp OR 'brexpiprazole':ti,ab,kw OR 'rexulti':ti,ab,kw OR 'opc-34712':ti,ab,kw) AND ('schizophrenia'/exp OR 'schizophrenia':ti,ab,kw OR 'major depression'/exp OR 'major depressive disorder':ti,ab,kw OR 'agitation'/exp OR 'alzheimer disease':ti,ab,kw OR 'adolescent'/exp) AND ('drug safety'/exp OR 'tolerability':ti,ab,kw OR 'drug efficacy'/exp OR 'pharmacokinetics'/exp OR 'health economics'/exp OR 'health care cost'/exp OR 'health care utilization'/exp OR 'market access':ti,ab,kw OR 'drug approval'/exp) AND ('india'/exp OR 'india':ti,ab,kw OR 'united states'/exp OR 'united states':ti,ab,kw)	Human; English language; Inception to August 2026
Scopus	August 27, 2026	TITLE-ABS-KEY("brexpiprazole" OR "Rexulti" OR "OPC-34712") AND TITLE-ABS-KEY("schizophrenia" OR "major depressive disorder" OR "Alzheimer disease agitation" OR "adolescents") AND TITLE-ABS-KEY("safety" OR "tolerability" OR "efficacy" OR "pharmacokinetics" OR "cost" OR "healthcare utilization" OR "market access" OR "regulatory approval") AND TITLE-ABS-KEY("India" OR "United States")	Document types: Article, Review; Language: English
Google Scholar	August 27, 2026	allintitle: brexpiprazole OR Rexulti (schizophrenia OR depression OR "Alzheimer" OR India OR US) supplemented by ("brexpiprazole" OR "Rexulti") AND ("market access" OR "claims" OR "cost" OR "pricing" OR "CDSCO" OR "FDA") AND ("India" OR "United States")	Sort by relevance; First 100 results screened
United States FDA Databases (Drugs@FDA & Orange Book)	August 28, 2026	("brexpiprazole" OR "Rexulti" OR "OPC-34712") AND ("prescribing information" OR "approval" OR "label" OR "Orange Book" OR "therapeutic equivalence")	Official US regulatory records
CDSCO Regulatory Portal (Govt. of India)	September 2, 2026	("brexpiprazole" OR "Rexulti") AND ("new drugs approved" OR "finished formulation" OR "subsequent approval")	Official Indian drug approval lists and notifications (2026)
National Health Financing Portals (NHSRC/MoHFW)	September 3, 2026	("National Health Accounts" OR "out-of-pocket expenditure") AND ("India")	Most recent official NHA release

Supplementary Table 2

**Table 7:** Quantitative summary of literature identification and screening

Source	Records identified	Duplicates removed	Records screened	Full texts assessed	Full texts excluded	Sources included
PubMed/MEDLINE	230	45	185	40	26	14
Embase	220	78	142	31	23	8
Scopus	157	62	95	22	18	4
Google Scholar	100	0	100	15	12	3
FDA/regulatory sources	5	-	5	5	3	2
CDSCO	4	-	4	4	3	1
Other official sources/reference-list screening	9	-	9	9	5	4
Total	725	185	540	126	90	36

Supplementary Table 3

**Table 8:** Composition of the evidence base (N = 36) MDD: major depressive disorder;  FDA: Food and Drug Administration; CDSCO: Central Drugs Standard Control Organisation

Evidence category	Description	Specific included references (from manuscript)	Source count (N = 36)
Randomized controlled trials (RCTs)	Pivotal double-blind, placebo-controlled phase 3 registration studies, randomized-withdrawal maintenance trials, and pediatric/dementia efficacy trials	Grossberg et al., 2020 [7]; Correll et al., 2015 [15]; Kane et al., 2015 [16]; Ward et al., 2025 [18]; Thase et al., 2015 (PYXIS) [20]; Thase et al., 2015 (POLARIS) [21]; McIntyre et al., 2025 [24]; Lee et al., 2023 [28]	8
Observational studies & real-world evidence	Retrospective commercial and Medicare claims analyses assessing costs, hospitalizations, mortality, and long-term open-label extension safety cohorts	Yan et al., 2020 (health care cost)[5] ; Seetasith et al., 2019 [8]; Zissimopoulos et al., 2025 [10]; Atkinson et al., 2025 [27]; Awasthi et al., 2025 [30]; Yan et al., 2020 (impact study) [31]	6
Systematic reviews & meta-analyses	Formal systematic reviews and network meta-analyses evaluating comparative efficacy, augmentation strategies, and tolerability in schizophrenia and MDD	Kishi et al., 2020 [6]; Khalid et al., 2026 [22]; McIntyre et al., 2026 [23]; Huhn et al., 2019 [25]	4
Regulatory documents & official government reports	Official regulatory prescribing information, patent and generic equivalence records, and official national drug-approval registries	US FDA Prescribing Information [3]; CDSCO List of New Drugs Approved [9]; US FDA Orange Book [32]; National Health Systems Resource Centre [35]	4
Epidemiological & mental health infrastructure reports	National and global burden of disease studies, population-based psychiatric prevalence estimates, psychiatric workforce density, and national health-financing/expenditure data	Solmi et al., 2023 [1]; Hegde et al., 2023 [33]; Ministry of Finance, Government of India, Economic Survey 2023-24 [34]; Garg et al., 2019 [36]	4
Preclinical pharmacology, clinical guidelines, & narrative reviews	Preclinical in vitro/in vivo receptor binding studies, professional society clinical guidelines, mechanistic reviews, clinical commentaries, and background clinical-trial context identified through reference-list screening	Keepers et al. (APA Guideline), 2020 [2]; Siwek et al., 2023 [4]; Fukuyama et al., 2022 [11]; Maeda et al., 2014 [12]; Frankel & Schwartz, 2017 [13]; Stahl, 2016 [14]; Crump et al., 2024 [17]; Rush et al., 2006 (STAR*D) [19]; Gowda & Nayok, 2026 [26]; Olivola et al., 2026 [29]	10
Total included sources			36
